# Supplementary material for: Are carbon emissions trading and green financial instruments synergistic? -Comprehensive quantitative research based on content analysis
Source: PLoS One. 2024 Mar 7;19(3):e0298601. doi: 10.1371/journal.pone.0298601 (PMC10919601; doi:10.1371/journal.pone.0298601)
Supplement: S1 Data — (ZIP) [file pone.0298601.s001.zip › Data/地级市Z_F工作报告.zip!/║■▒▒/╥╦▓2╩╨/└┤╫╘╣1⁄2╓┌║┼í╛┬φ┐╦╩2╛▌═°í┐_2015.docx]

各位代表：

　　现在，我代表市人民政府向大会报告工作，请予审议，并请各位政协委员和列席人员提出意见。

　　一、2014年工作回顾

　　过去的一年，面对错综复杂的宏观环境和阶段性特殊困难，我们全面贯彻落实省委省政府和市委的决策部署，以党的群众路线教育实践活动为契机，稳神定气、克难攻坚，全市经济社会发展实现稳中有进、质效同步，基本完成市五届人大五次会议确定的目标任务。

　　（一）稳增长、争进位，综合实力不断提升。坚持把保持经济持续健康发展作为首要任务，综合施策、精准发力，主要经济指标增速继续高于全省全国。实现生产总值3132.2亿元，增长9.8%；地方公共财政预算收入271.5亿元，增长31.6%；固定资产投资2471亿元，增长22.1%；社会消费品零售总额964.5亿元，增长13.3%；外贸进出口27亿美元，增长14.8%；城镇居民人均可支配收入25025元，增长9.6%；农村居民人均可支配收入11837元，增长13.2%。县域经济竞相发展。宜都成为全省第二个全国县域经济百强县（市）。宜都、夷陵、枝江、当阳在全省县域经济分类考核中位居前列。8个县（市、区）地方公共财政预算收入超过10亿元，其中2个过20亿元、2个过30亿元。9个县（市、区）城镇居民人均可支配收入超过2万元，8个县（市、区）农村居民人均可支配收入超过1万元。

　　（二）转方式、调结构，产业升级步伐加快。坚持把结构调整作为主攻方向，利用市场机制倒逼转型升级。重点产业发展提速。精细化工、食品生物医药、先进装备制造、新材料产业总产值占全市工业比重达到78%，精细化工、食品生物医药产业产值均突破1500亿元，先进装备制造业产值突破1000亿元，新材料、文化旅游、现代物流业产值保持两位数增长。新兴产业不断壮大。电子信息、新能源、节能环保产业发展加快。高新技术产业增加值475.6亿元，占生产总值比重提高3个百分点。三次产业结构优化。规模工业增加值增长11.2%。净增规模工业企业181家、产值过亿元企业79家。2家企业入选中国企业500强，4家企业入选中国民营企业500强，15家企业入选湖北民营企业100强。第三产业增加值占生产总值比重提高1.4个百分点。三峡旅游新区建设加快推进。三峡大坝景区实行门票免费，屈原故里文化旅游区晋升国家5A。全年接待游客4085万人次，旅游总收入336亿元，分别增长23%、29.2%。新增限额以上商贸企业超过200家。广发、光大银行在宜设立分行。新设立村镇银行3家、小额贷款公司3家。金融机构存贷款余额分别达到2583亿元、1968亿元。六大特色农业产业占农业总产值比重达到82.8%。农产品加工产值达到1600亿元。发展后劲持续增强。招商引资实际到位资金1239亿元，增长33%。惠科电子、东土科技、1号店等国际国内龙头企业落户宜昌。我市跻身“中国最佳营商环境十大城市”。投资亿元以上在建项目1126个，增加264个。67个省市重点项目超额完成年度投资计划，南玻平板显示器、宜昌汽车产业园、人福药业出口生产基地等一批重点项目竣工或投产。宜昌高新区引进投资10亿元以上项目17个，开工建设重点产业项目63个。落实三峡对口支援资金99.6亿元，上海对口支援宜昌合作领域进一步拓展。争取到位三峡后续工作项目补助资金10.6亿元。质量强市创建深入推进。我市被评为“2014年度全国质量魅力城市”。新增中国驰名商标4件，达到39件。新增湖北名牌产品12个。稻花香酒业公司获“长江质量奖提名奖”。资本市场多元发展。新增上市企业1家、“新三板”挂牌企业2家、“四板”挂牌企业6家。上市企业再融资12.1亿元，本地企业债券融资74亿元。

　　（三）抓改革、促创新，活力动力更加强劲。坚持把改革创新作为根本动力，努力使市场在资源配置中起决定性作用，同时更好地发挥政府作用。主动学习对接上海自贸区，确立“1＋6＋4＋5”对接格局，市场准入、简政放权、投资制度、社会信用、行业监管、综合执法等改革加快推进。在全省率先实行“先照后证、三证合一”，新增市场主体8.9万家，民间投资、第三产业投资分别增长30.4%和21.4%。全市公共信用信息平台框架基本形成。市级国有投融资公司改革重组有序推进，全年融资220.6亿元。市属国有企业改革发展步入快车道。城区城管体制进一步深化，大城管格局基本形成。财政预算、投入、绩效管理改革富有成效。农村土地承包经营权确权登记颁证试点和农村集体建设用地、宅基地使用权确权登记工作顺利推进，全市土地流转面积达到75万亩，新增农民合作社722家、登记注册家庭农场588家。完成食品药品监管体制改革，全国最佳食品安全放心城市创建扎实推进，我市被评为“2014中国十佳食品安全城市”。政府机构改革、事业单位分类改革、医药卫生体制改革进展顺利。创新要素加快聚集。新认定国家高新技术企业60家、国家级孵化器1家、省级工程技术研究中心11家、市级工程技术中心10家。新增孵化器面积20万平方米。新增发明专利授权276件、高新技术产品55个。安琪酵母产业化项目获国家科技进步二等奖。中国科协在宜设立“海智计划”工作站。新建院士专家工作站18家、博士后创新基地3家。新增省级以上专家11人。全市企业技能人才评价、非公企业职称评审工作全面推进。我市获批创建全国生态文明建设先行示范区、国家新能源示范城市、国家电子商务示范城市，成为全国产学研合作创新示范基地、国家信息惠民试点城市、国家食品安全城市创建试点城市、全国养老服务业综合改革试点城市、全国基层卫生综合改革重点联系市。

　　（四）重统筹、优功能，大城建设提速增效。坚持把中心城区作为龙头来抓，统筹推进城市现代化、乡村田园化、城乡一体化。宜昌新区新建续建项目202个，完成投资310亿元。开工建设城市道路121公里，竣工通车45公里，至喜长江大桥工程进度完成70%，城区快速环网雏形初显。东山大道BRT改造工程加快实施，项目被亚洲银行评为国内唯一可持续交通项目最佳案例。开工建设大型公建项目18个，市规划展览馆、市博物馆主体结构基本完工，市奥体中心进入主体施工阶段。市规划展览馆被评为全省高星级绿色建筑示范创建项目。续建、新建安置房518万平方米，竣工69万平方米。磨基山公园一期和东山公园一期建成开放，滨江公园景观实现提档升级。中央商务区滨江项目、点军生态新城等城市综合体建设加快推进。旧城更新步伐加快。环城南路、白沙路等棚户区改造项目全面启动，争取国家开发银行棚户区改造专项贷款127亿元。完成建筑综合整治项目172个，安装景观灯9万余盏，绿化开工面积260万平方米，定植绿化大苗6.32万株。城区运河整治主体工程历时三年圆满完成。完成13个重点电网建设工程。增加城区公共停车位1060个。市域规划实现全覆盖。我市连续六届摘取全省城乡规划建设管理“楚天杯”桂冠。城区建成区面积达到152.6平方公里。全市城镇化率达到55.65%。交通体系日趋完善。完成交通投资110亿元。宜巴、保宜高速建成通车，新增高速公路204公里。建成一级公路110公里，改造二级公路150公里，硬化农村公路900公里。三峡枢纽港区建设有序推进，宜昌长江干支流岸线规范整治全面启动，三峡现代物流中心基础设施建设项目获2亿美元世界银行贷款支持，三峡翻坝物流园建设上升为省级战略，白洋港一期、茅坪港二期加快建设。宜渝铁路开通动车。三峡机场被评为国际航空服务四星机场，年旅客吞吐量突破100万人次。城区公交新增12条线路，城际公交开通2条线路。村镇建设力度加大。基础设施建设投入13亿元。90%的村庄完成环境整治任务，63%的农村垃圾实现无害化处理。完成清洁能源入户工程2.5万户。治理水土流失51.22平方公里。建设末级渠系650公里，除险加固中小型水库41座。生态建设扎实推进。完成《宜昌市环境总体规划》编制。淘汰城区燃煤锅炉25台，关闭安能、美联热电等小火电机组8台、土砖窑39座。机动车环保标志管理全面实施，淘汰黄标车3.2万台。黄家湾垃圾填埋场提前封场。五峰县城避险迁建完成主体工程。3个村被纳入国家避险解困试点。市级以上生态村镇达到186个。6个村镇上榜“荆楚最美村镇”。全市单位生产总值能耗、地耗分别下降4.8%和8.43%。补充耕地9750亩，连续12年实现占补平衡。4家企业入选国家级绿色矿山试点单位。

　　（五）惠民生、促和谐，人民生活明显改善。坚持把民生幸福作为不懈追求，深入推进五大民生工程，让人民共享改革发展成果。全市财政民生支出增长23.5%，占地方公共财政支出总额的69.5%。居民消费价格涨幅回落至1.2%。城镇新增就业人员11万人，组织农村劳动力转移就业10.9万人, 城镇登记失业率降至2.23%。社保扩面17.4万人次，城乡居民基本养老保险适龄人员参续保缴费率达到100%。职工省内异地就医、居民大病保险实现即时结算，全市医疗保险“一卡通”平台正式启用。新农合参合率99.8%，参合农民住院费用报销比例进一步提高。城乡居民养老金、被征地农民养老金、企业退休人员养老金和城区环卫工人工资待遇得到提高。城乡低保对象、优抚对象和特殊困难群体救助保障水平稳步提升。我市蝉联“中国城市慈善百强”。建成城市社区养老服务中心207个、农村老年人互助照料活动中心708个。保障性住房和棚户区改造住房开工65188套，建成23301套，分配入住14383套。完成农村危房改造10962户。解决农村饮水安全26万人。30个特困村脱贫攻坚行动全面实施，4.5万贫困人口稳定脱贫。兴山、远安被纳入我省秦巴山片区扶贫规划范围。

　　公共文化服务体系建设全面启动。完成15个街办文化站和342个村文化室改扩建。成功举办屈原故里端午文化节、第四届中国宜昌长江钢琴音乐节暨宜昌首届艺术节。承办国家和省体育赛事31项。我市选手在仁川亚运会上取得4金1铜好成绩。3个项目入选中国体育旅游精品项目。歌曲《江河恋》获得全国“五个一工程”奖，《中华鲟历险记》获中国最佳动画电视入围奖。我市被命名为“中国诗歌之城”、“中国钢琴之城”。全民阅读指数测评结果位居全省同等城市第一。完成宜昌民俗志、县级综合志等志书编纂工作。

　　第一期学前教育三年计划全面完成，新建、改扩建幼儿园94所，学前三年毛入园率提高到86%。我市成为全省义务教育均衡发展整体通过国家验收的唯一地级市。职教园一期工程投入使用。职业教育质量稳步提升。青少年实践教育基地、市一中及夷陵中学新校区等项目前期工作有序推进。三峡大学整体进入一本招生，跻身“全国高校就业50强”。资助贫困学生12.8万人次、1.46亿元。宜昌0～3岁儿童早期教育纳入联合国儿童基金试点项目。城乡医疗卫生服务体系不断完善，村卫生室公有化建设强力推进，乡村医生定向委培三年计划全面启动。市中心医院、市一医院门急诊大楼等项目加快推进。我市顺利通过全省健康城市考核。在全省率先实现独生子女年老父母计生奖励全覆盖。

　　巩固“一本三化”服务体系，我市在全国率先实现城乡网格化管理全覆盖，在全省率先实现农村光纤“村村通”。积极创建全国第四届文明城市，在中央文明委考核中获得优异成绩。50人入选“中国好人榜”、“湖北好人榜”和“荆楚楷模”。7个单位被评为全国和谐社区建设示范城区、示范街道、示范社区。“幸福村落”创建覆盖率达到40%。村委会换届选举全面完成。法治宜昌建设深入推进，办理法律援助案件2103件、法律援助事项19221件。平安宜昌建设成效明显。安全生产形势总体稳定。

　　（六）转作风、提效能，自身建设全面加强。扎实开展党的群众路线教育实践活动，深入贯彻落实中央关于改进工作作风、密切联系群众的“八项规定”精神，切实解决“四风”突出问题，取得初步成效。清理规范行政权力，全面公开市级权力清单。取消和调整市级行政审批事项97项。优化行政审批流程，推行并联审批、网上审批，审批时限压缩75%。政务服务中心标准化、信息化建设稳步推进。出台行政机关首受责任制等51项制度，实施10项专项整治，开展政风行风评议和“电视问政”活动。全市“三公”经费下降28%，会议、文件、评比活动数量减少23%。坚持“力度统一论”，改革发展和反腐倡廉工作成效显著。行政监察和审计监督进一步加强。自觉在人大的法律监督和工作监督、政协的民主监督下开展工作，定期向市人大报告工作、向市政协通报工作，按期办复人大议案和建议273件、政协建议案和提案567件。

　　2014年，民族宗教、援藏援疆、统计、信访、气象、对台、外事、侨务、粮食、供销、档案、保密等工作迈上新台阶；国防动员、民兵预备役、双拥创建、人民防空、防震减灾、消防等工作取得新成效；妇女儿童、青少年、老龄、残疾人等事业实现新发展。全面完成第三次经济普查。服务三峡工程安全运行成效明显，三峡电站年发电量跃居世界首位，三峡船闸连续11年实现安全高效运行。

　　各位代表！在全国全省经济增速放缓、下行压力持续加大的背景下，我市经济运行呈现调速不减势、量增质更优的良好态势，发展活力和后劲进一步增强，省委省政府给予充分肯定。成绩来之十分不易，这是省委省政府和市委正确领导的结果，是市人大、市政协大力支持的结果，是全市人民团结拼搏的结果。在此，我代表市人民政府，向全体人大代表、政协委员，向广大干部群众、离退休老同志，向各民主党派、工商联、无党派人士、各人民团体，向驻宜部队官兵、中省驻宜单位，向所有关心、支持和参与宜昌建设的海内外朋友，表示诚挚感谢！

　　我们也清醒地认识到，当前宏观环境依然复杂，实体经济运行仍然困难，产业整体实力不够雄厚，扩大总量与提升质量的双重压力仍然较大；自主创新能力还不够强，资源环境约束加剧，转型升级任务艰巨；民生工作还存在不少薄弱环节，公共服务资源配置还不够均衡，城乡居民增收难度加大，社会治理面临许多新的挑战；少数部门和干部服务意识不强、执行落实不力的问题仍然存在。对此，我们一定认真对待，采取有力措施，切实加以解决，决不辜负人民的厚望。

　　二、2015年工作重点

　　今年是全面深化改革的关键之年、全面推进依法治国的开局之年，也是全面完成“十二五”规划任务的收官之年。综观国际国内发展大势，世界经济缓慢复苏，国内经济长期向好的基本面没有改变，特别是党的十八届三中、四中全会所释放的制度红利，长江经济带新一轮开放开发、长江中游城市群建设、三峡后续工作规划实施、省域副中心城市建设等重大战略机遇叠加，将为我市创造良好的发展环境。尤为重要的是，经过多年发展，宜昌以优势特色产业发展为主导的新型工业化进程、以中心城市建设为引领的城乡发展一体化进程、以沿江突破为特征的对外开放进程、以全国生态文明建设先行示范区创建为抓手的生态文明建设进程不断加快，内在活力和竞争优势日益增强。我们完全有基础、有条件、有能力开创全面深化改革的新局面，迈出现代化建设的新步伐！

　　今年政府工作的总体要求是：全面贯彻党的十八大和十八届三中、四中全会精神，认真落实省委十届五次、市委五届五次全会决策部署，坚持法治引领、改革创新，实施高质量可持续发展核心战略，主动适应经济发展新常态，以更高标准、更大作为，坚定不移调结构、转方式，狠抓改革攻坚，突出创新驱动，加强民生保障，推动经济提质升级增效，促进社会和谐稳定，全面完成“十二五”规划任务，加快建设现代化特大城市，努力向中部地区同等城市首位迈进。

　　全市经济社会发展的主要预期目标是：在优化结构、提高质量的基础上，生产总值增长10%以上，规模以上工业增加值增长11.5%以上，地方公共财政预算收入增长12%以上，固定资产投资增长20%以上，社会消费品零售总额增长13%以上，外贸出口增长10%以上，城乡居民收入增速与经济增长同步，居民消费价格涨幅控制在3.5%以内，城镇登记失业率控制在4.2%以内，全面完成省下达的节能减排任务。

　　围绕实现上述目标，重点做好五个方面的工作。

　　（一）更大力度推进改革开放，激发体制机制新活力

　　深化重点领域改革。进一步解放思想，以学习对接上海自贸区为突破口，坚持问题导向，着力破解制约发展的体制机制障碍。进一步深化行政审批制度改革，继续推进简政放权。向社会集中公布行政权力目录清单、涉企收费清单、行政审批并联操作流程图。推进与行政审批相关的中介机构规范化、市场化改革。全面推行“先照后证、三证合一”，推进企业登记制度便利化。建立健全事中事后监管机制。加快社会征信体系建设，建成公共信用信息平台。深化财政改革，加快建立现代财政制度。完善政府全口径预算，建立跨年度预算平衡机制，继续推进预决算公开，推进财政资金竞争性分配和预算绩效改革，加大统筹使用财政资金力度。进一步完善事权与支出责任相匹配的财政体制，促进扩权强区。推进政府向社会组织购买公共服务。严格政府债务、政府投资项目管理，清理规范税收等优惠政策。深化金融改革，积极引进银行、证券、基金、信托等金融机构，加快发展地方金融、民营金融、小微金融。多渠道推动股权、债券、项目融资，支持企业扩大直接融资规模，探索推进农村土地承包经营权、农民住房财产权融资。创新重点领域投融资机制，鼓励社会投资。更大力度促进非公有制经济加快发展。以优化国有企业产权结构、完善法人治理结构为重点，深化国有企业改革，大力发展混合所有制经济。完成市级国有投融资公司改革重组。推进土地承包经营权、农村集体建设用地使用权和农户宅基地确权登记工作。加快推进不动产登记。深化文化体制改革，促进文化事业全面繁荣、文化产业快速发展。深化教育综合改革，落实立德树人根本任务。深化医药卫生体制改革，加快县级公立医院和基层医疗卫生机构综合改革，建立医疗卫生机构运行新机制。进一步深化价格体制机制改革。稳步推进机关事业单位养老保险制度改革。完成党政机关公务用车制度改革。统筹抓好政府机构改革、事业单位分类改革等其他各项改革。

　　提升招商引资质效。突出产业招商，强化专业招商，推进以商招商、网络招商、代理招商，形成政府推动、园区带动、企业主导、中介服务的招商新格局。围绕战略性新兴产业、先进制造业、现代服务业，重点引进一批关联度高、带动力强的重大项目、高端项目。围绕六大千亿产业和特色优势产业，强化龙头延链、配套补链，着力引进一批配套型、服务型、延链型项目。围绕本地优势资源，做好项目储备、策划、包装、推介，力争一个特色优势资源形成一个产业链。紧盯世界500强、全国500强、行业100强、大型上市企业，实行高位对接，推动在宜设立制造中心、生产基地、研发中心、企业总部和功能性机构。引导本地企业与境内外大企业、大财团进行战略合作。鼓励支持现有企业技改和新上项目，加快转型升级。坚持大小兼顾、内外并重，力争全年招商引资实际到位资金1200亿元，直接利用外资3.5亿美元，新开工亿元以上招商项目370个。严格规范招商引资项目评审，加强绩效评价，切实提升招商引资工作水平。探索园区市场化运作机制，鼓励有条件的园区采取股权合作、引进第三方专业企业托管等模式进行开发管理。深化与上海自贸区、武汉东湖高新区等地招商协作。

　　扩大对内对外开放。抢抓长江经济带开放开发重大战略机遇，深入推进沿江突破，着力打造沿江万亿经济带，加快把宜昌建设成为全国重要的综合交通枢纽和现代物流中心、东部沿海产业转移示范基地、三峡城市群中心城市。加强与上海、武汉、重庆、南通等沿江城市联系，以港口合作为纽带，促进产业协作、要素流动与共同发展。加强与长三角、珠三角、环渤海等发达地区合作，积极承接沿海产业转移。推进三峡城市群在交通、旅游、环保、科技、教育、文化、市场、基础设施等方面有效对接，成为长江中游城市群和成渝城市群之间的重要支撑点。深入实施三峡后续规划和新一轮对口支援三峡库区合作规划，加强与支援方合作，争取对口支援到位资金85亿元。深化与长江三峡集团、中建三局、葛洲坝集团、武钢集团等中省企业联系，加快在旅游、物流、交通、城建、钢材加工、载电工业等领域推进战略合作。鼓励县市强化与发达地区的跨区域合作，支持山区、库区县到沿江区域发展“飞地经济”。积极创新投资贸易、综合监管等制度，加快推进宜昌保税物流中心申报建设。积极参与长江经济带海关区域通关一体化，优化“大通关”环境。主动融入汉新欧、渝新欧跨国物流通道。加强国际友好城市交往。鼓励企业发展电子商务，扶持企业开拓国内外市场，争创具有国际竞争力的出口品牌。支持企业对外投资，开展劳务合作，拓展国际发展空间，努力提高宜昌经济外向度。

　　（二）更大力度推进转型升级，培育经济竞争新优势

　　积极扩大有效投资。集中各方面智慧和力量，科学编制“十三五”规划。认真谋划一批对经济发展和结构调整全局带动性强的重大工程，对推进社会建设、生态环保、改善民生作用显著的重大项目，对解决突出矛盾、增进公平效率有力有效的重大政策，为补短板、增后劲、促均衡、上水平提供支撑。把握国家“十三五”政策和方向，找准与宜昌的最佳结合点，策划实施一批技术领先的先进制造业和战略性新兴产业项目，一批产业结构升级的现代服务业项目，一批打基础、管长远的重大基础设施项目，力争有更多项目进入国家、省规划。继续开展“重大项目建设年”活动，突出先进制造、现代服务、基础设施、大型公建等重点领域，加快推进惠科显示器、兰台科技、贝因美婴童产业园、华强科技产业园、广州无线电集团宜昌高端金融电子产业园、生态智慧产业新城、通航产业园等100个省市级重点项目建设，确保全年完成投资3000亿元。创新重大项目推进体制机制，切实解决好项目建设中的融资、用地、拆迁等实际问题，营造一流的投资环境，促进项目早落地、早开工，使储备、策划、签约项目尽快转化为建设项目。

　　着力优化产业结构。充分发挥产业资金的导向作用，引导社会资本投向重点产业领域，做大做强六大千亿产业。确保规模以上工业产值突破5500亿元，新增规模以上工业企业130家以上。推进工业加快转型升级，增强市场竞争力和发展后劲，确保精细化工、食品生物医药产业产值均突破1700亿元，先进装备制造业突破1100亿元。加大规划引导和政策支持力度，优先发展新一代信息技术、新材料、新能源、节能环保等战略性新兴产业，抓好国家、省级试点示范，尽快把新兴产业培育成支柱产业，确保新材料产业产值突破700亿元。加快实施城区老工业基地整体搬迁改造，推动工业化和信息化融合发展，促进制造业向研发设计和营销服务两端延伸，促进传统产业提档升级。推动落实国家促进中小微企业发展的政策措施，营造企业发展的良好环境。

　　加快转变农业发展方式，按照“统筹融合、特色引领、转型升级、创新驱动”的思路，努力建设现代农业强市。着力推进特色产业转型升级，坚持绿色、生态、安全、高效原则，进一步聚焦重点特色产业，重点实施“柑桔品质提升”行动，优化结构、壮大规模、提升品质、开拓市场；着力实施“提升绿茶、振兴红茶”战略，重点加快推进红茶品牌创建、科技攻关和茶叶绿色防控工作；大力实施“畜牧业跃升”行动，推进牛羊产业发展，优化产业、品质、种养结构，加快建设中部地区畜牧强市。突破性发展农产品精深加工，继续组织实施“百亿企业”成长工程、农产品加工示范园区创建工程和精品名牌创建工程，支持稻花香集团在全省率先冲刺500亿产业集群，努力构建农产品加工业发展新优势，力争农产品加工产值与农业总产值之比达到2.5:1以上。全力推进农业科技创新，重点建设土老憨柑桔、水产品精深加工，屈姑脐橙精深加工，萧氏、采花茶叶精深加工，一致魔芋精深加工等农业科技产业链。大力培育新型农业经营主体，加快发展家庭农场、专业大户、农民合作社、龙头企业、农业社会化服务组织，积极引导农业经营主体规范化发展、标准化创建，促进农业适度规模经营。

　　加快发展现代物流、金融服务、商务服务、科技服务、信息服务等生产性服务业，推进服务业集聚区建设。抓住国家规划建设三峡翻坝综合运输体系机遇，加快建设三峡枢纽港区，重点推进白洋港一期、茅坪港二期、三峡翻坝物流园建设，力争现代物流业产值突破1000亿元。优化金融生态环境，提升金融服务水平，促进金融业稳定发展。加强企业上市培育工作，力争新增上市企业1家。加快发展电子商务、服务外包、快递物流、大数据等以互联网为基础的新型业态。加快发展总部经济和楼宇经济。抓紧实施千亿文化旅游产业三年行动计划，确保文化旅游业产值突破600亿元。促进旅游业与文化、商贸、工业、农业、体育等产业融合发展，确保全年接待游客4500万人次、旅游总收入450亿元以上。加快推进三峡旅游新区建设，积极创建三峡国家级旅游度假区和旅游名镇名村。支持三峡大瀑布旅游区创建国家5A景区。重点推进关公文化旅游城、百里荒生态农业旅游度假区、磨基山旅游公园等项目竣工营运。加快推进平湖半岛旅游综合开发、清江画廊景区整体升级、玉泉山风景区整体开发和柴埠溪旅游度假区、龙泉古文化商业街等重点项目建设。加快创建全国旅游标准化示范城市，重点建设城市一、二级游客集散中心。办好第六届中国长江三峡国际旅游节和第五届中国宜昌长江钢琴音乐节。抓好钢琴文化产业园等十大产业园区建设。高度重视文化创意产业，促进文化创意、设计服务与相关产业融合发展。推进宜昌文化创意产业中心建设。推动传统媒体与新媒体融合发展。适应消费结构升级，积极发展健康服务、养老服务、教育服务、社区服务等新兴服务业，增强消费对经济增长的拉动力。确保新增限额以上商贸企业100家。

　　大力实施创新驱动。发挥自主创新在转型升级中的引领和支撑作用，着力创建国家创新型试点城市和质量强市示范市，积极推进智慧城市建设。创新科技投入机制，强化财政投入导向性作用，激励企业加大研发投入，吸引各类资本投入科技型企业。大力发展高新技术产业，实施中小企业创新成长工程，确保新增国家高新技术企业40家以上，高新技术产业增加值突破550亿元，占生产总值比重提高1个百分点以上。加快重点行业企业技术改造步伐，实施50个重大技术改造示范项目。深化产学研合作，力争在电子信息、新材料、精细化工、先进装备制造、生物医药等领域的关键技术上实现突破。围绕六大千亿产业、战略性新兴产业，构建技术公共服务、技术成果交易、标准创新、融资服务等创新服务平台。规划建设“宜昌智谷”，搭建创新创业公共服务和人才生活服务平台。新增省级以上科技研发平台6家，新建市级工程技术中心、重点实验室8家。加快推进三峡检验检测中心建设。加强高新技术园区、科技园区、创业园区建设，新建孵化器面积20万平方米以上，在孵企业总数达到400家，推进科技成果产业化，着力培育一批小微型科技企业。加强知识产权运用与保护，加快建设国家知识产权示范城市。加快推进人才强市改革试验区建设，深入实施“宜昌英才工程”，大力引进以高端产业人才和创新创业人才为重点的各类高层次人才和团队，大力培育企业家、急需紧缺专业技术人才和产业技能人才。激发全民创造、创新、创业活力，形成大众创业、万众创新的生动局面。

　　（三）更大力度推进城乡统筹，彰显城市发展新魅力

　　加快推进宜昌新区建设。坚持用后现代理念、市场化手段建设新区，实施343个城建项目，强力推进基础设施、大型公建、景观园林、安置房建设，确保全年完成投资632亿元。以快速路网引领新区建设，进一步拓展城市骨架。加快江城大道、西陵二路等快速路建设，逐步形成以至喜、夷陵、伍家岗、宜昌长江公路大桥等四座长江大桥为联接的“内中外三级”快速环网。力争今年内环闭合、2016年外环闭合、2017年中环闭合。抓好点军大道二期、峡州大道二期、花溪路等续建道路建设。积极推进东站片区、城东片区、奥体片区、唐家湾片区、五龙片区等重点开发区域建设。完成市音乐厅、大剧院、科技馆、科教城、档案馆、电视观光塔等大型公建项目前期工作。确保市博物馆、市奥体中心、市规划展览馆年内基本建成。加快建设中央商务区滨江项目、娃哈哈购物广场等城市综合体。科学编制中心城区生态景观规划，加快建设城区“六大水系、八大公园”，重点实施磨基山、卷桥河、城东、东山、柏临河等公园项目，抓好滨江公园中段升级改造，建设绿道80公里。延续城市历史文脉，积极推进环城南路历史文化风貌街区建设。

　　着力做大做强城区经济。加快城区工业转型发展，调整园区运作模式，实行宜昌高新区“一区统筹”、工业园区“分区实施”。完成高新区空间布局规划编制，统筹布局产业项目。推动东山园区转型升级。盘活猇亭园区存量要素资源，提升园区承载能力。加快电子信息产业园白马路、双十路、天台路等基础设施建设，拉开园区骨架，尽快形成核心区。改进生物产业园、白洋工业园项目落地条件，进一步提高投资强度和产出效益。确保新引进项目80个，完成投资400亿元。支持各区引进项目落户宜昌高新区，支持设立区级工业发展专项资金。支持西陵区、伍家岗区以总部楼宇、商贸物流、科技孵化、生物医药、金融服务为重点，加快发展都市工业和现代服务业。支持点军区以发展电子信息、文化旅游、都市农业为重点，加快建设滨江生态新城区、全省绿色示范区。支持猇亭区以精细化工、先进装备制造、新材料产业等为重点，积极发展循环经济、临港和临空经济。支持夷陵区争创全省城乡统筹发展示范区，加快与中心城区深度融合。

　　推动县域发展提档升级。鼓励县市加快建设特色园区，不断壮大产业实力。加快推动产业、项目、环保、基础设施市县对接，实现融合发展。支持宜都保百强、争进位。支持枝江、当阳冲刺全国百强。支持远安争创“绿色湖北、美丽荆楚”典范。支持长阳建设都市后花园、低碳示范区，秭归建设长江经济带桥头堡，兴山建设山区生态经济强县。支持五峰建设武陵山科学发展示范区、绿色文明发展示范县。发挥安福寺、龙泉等11个新型城镇化建设试点镇示范作用，推动县域重点镇优先发展、中心镇加快发展、其他乡镇竞相发展，建设一批工业主导型、商贸带动型、现代农业型、旅游休闲型小城镇，吸引农业转移人口就地就近城镇化。

　　促进基础设施互联互通。全面实施宜昌新型城镇化规划，积极推进以人为核心的新型城镇化。优化城镇空间布局，统筹推进中心城区、县市城区、重点城镇和新型社区建设。优先发展公共交通，加快中心城区与周边县市基础设施互联互通。完善城乡路网系统，开工建设夜明珠路、江南二路等道路，加快东山大道延伸段、中南路延伸段、桔乡路等道路建设，全面完成东山大道BRT改造。积极推进社会停车场建设，新增公共停车位 1000个。加快推进三峡综合交通运输体系建设，完成交通投资110亿元。加快推进宜岳高速、宜张高速宜昌段建设，加快宜昌至来凤高速、江北翻坝高速等项目前期工作。推进三峡专用公路对社会开放。建成至喜长江大桥。加快建设香溪长江大桥。力争开工建设伍家岗长江大桥、白洋长江大桥。启动建设宜昌至黄花一级公路，完成当阳至枝江、318国道万城大桥至云池段等一级公路建设。建设一级公路100公里、二级公路200公里、县乡公路80公里、通村水泥路1000公里。改造完成6个县级客运站，加快实施行政村“村村通客车”工程和农村公路安保工程。加快推进三峡枢纽港区与沿江园区的通道建设，扩大港口运输服务覆盖范围。加快推进江北、江南翻坝铁路和宜昌至常德客运专线前期工作，确保紫云地方铁路全线建成通车。积极推动城市轨道交通建设。支持三峡机场改扩建，增加运输航线，拓展航空服务。

　　（四）更大力度推进生态建设，展现美丽宜昌新面貌

　　加大环境治理力度。完善大气污染联防联控联治体系，强化工业废气、机动车尾气、城市扬尘、油烟污染、秸秆焚烧、垃圾焚烧等综合治理。加快淘汰黄标车，主城区全面禁止黄标车通行和烟花爆竹燃放。加快推进城区清洁能源改造。加强对重点污染排放企业在线监控。推行水污染防治“河长制”。加快实施三峡库区、长江宜昌段水污染防治项目。重点推进黄柏河流域生态保护，统筹抓好清江、沮漳河、香溪河等流域及桥边河、柏临河、玛瑙河等中小河流治理工作。加快实施沙河综合整治工程。开展主要流域断面水质监测和考核。严格集中式饮用水源地保护。依法取缔水库投肥养殖和江河湖库围栏围网养殖。加强城区污水收集管网建设和沿江排污口治理。加快推进沙湾、田家河等城镇污水处理厂及配套管网建设。加强长江等重点区域地质灾害防治，抓好矿产采空区生态环境恢复治理。加大采石采砂监管力度。治理水土流失面积50平方公里。深入开展生态县、乡、村创建活动，加强畜禽养殖污染、土壤污染、农村生活垃圾治理，减少农业面源污染，提升农村环境综合整治实效。建成100个省级宜居村庄、164个特色村庄。建设高标准基本农田40万亩。改造末级渠系500公里。完成58个农村水电增效扩容改造项目。严格执行环保责任追究制度和环境损害赔偿制度，严厉打击环境违法行为。

　　改善城乡人居环境。坚持建城市必须重生态、管行业必须管环保，确保通过国家环保模范城市复核，加快创建国家生态文明建设先行示范区和国家生态园林城市。严格执行宜昌市环境总体规划，实施环境分级管控。继续推进城区生态建设“十大工程”。实施最严格的耕地保护、水资源管理等制度。划定第二批永久性保护山体及水域。加快推进绿化美化行动，确保在全省率先实现全域绿色全覆盖，建设全国林业综合治理示范区。切实加强天然林保护，扎实推进新一轮退耕还林。突出抓好交通干道沿线、长江岸线、产业园区等重点区域绿化美化工作。完成植树造林15万亩，城区定植绿化大苗3万株。调整完善土地利用总体规划。全面完成低丘缓坡地综合开发利用试点，加快推进城乡建设用地增减挂钩、城镇低效用地再开发、工矿废弃地复垦利用试点。启动新一轮国土资源节约集约模范县市创建活动。大力推进节约集约用地和闲置低效用地处置，确保全市单位生产总值地耗下降6.99%。加快推进旧城更新，重点抓好西陵一路、西陵二路、中南路和夷陵广场、五一广场、九码头、平湖、红光港机“三线五片”棚户区改造。完成建筑综合整治项目200个，启动建筑楼顶整理工作。推动县市城镇开展城市整理。继续抓好重要建筑、公共绿地等夜景灯光建设。完善城市管理组织体系，延伸城市管理考评范围，提高精细化管理水平。加强城区地下管线管网建设管理。进一步抓好餐厨废弃物资源化利用。建设一批环卫设施。力争城区生活垃圾无害化处理率达到96%、道路机械化清扫率达到65%。

　　强力推进节能减排。完善节能预警调控机制，加快节能技术、产品的开发和推广应用，重点抓好工业、交通、公共机构等领域节能降耗工作。确保全市单位生产总值能耗下降4%。大力发展节能绿色建筑，新增可再生能源建筑应用面积157万平方米、绿色建筑100万平方米。深入开展企业能效对标活动，推广合同能源管理、余热余压利用等节能措施，加快实施污染物减排项目。继续推进猇亭园区循环化改造试点，加快建设一批生态工业园区和可再生资源基地。

　　（五）更大力度推进民生改善，回应人民群众新期盼

　　深入实施民生工程。加快推进城乡居民收入倍增工程，持续增加居民收入。全面落实就业创业扶持政策，继续完善城乡一体、资源共享的公共就业服务网络，持续开展城乡劳动者就业创业培训，重点抓好高校毕业生、下岗失业人员、农村富余劳动力、城镇困难人员等群体就业，大力促进创业带动就业。确保城镇新增就业7.7万人。积极创建和谐劳动关系，切实保障劳动者合法权益。推进食品药品网格化监管区域全覆盖，治理“餐桌污染”，抓好小作坊、小摊贩、小餐饮、小食品店专项整治，强化示范创建，加强综合执法，规范市场秩序，加快建设全国最佳食品安全放心城市。推进价格网格化监管。加大精准扶贫力度，继续实施片区扶贫攻坚、30个特困村脱贫攻坚行动和86个村扶贫开发整村推进，确保全市减贫4万人。扎实做好移民、残疾人同步小康工作。积极完善各项社保政策，努力提高待遇标准。完善新农合大病保障机制，加快推进新农合市级统筹。扩大社会保险覆盖面，确保城乡居民基本养老保险适龄人员参续保缴费率90%以上。实施棚户区改造39438套、开工建设公租房6288套。改造农村危房8000户。完成“十二五”农村饮水安全工程建设任务。确保社会救助、优抚安置、社会福利等保障标准与城乡居民平均生活水平同步提高。为困难群众和农民工提供法律援助。完善社会基本养老服务体系，加快发展居家养老服务，力争城市社区养老服务中心全覆盖、农村老年人生活互助照料中心覆盖率达到50%，积极做好全国养老服务业综合改革试点工作。

　　推进社会事业进步。大力培育和践行社会主义核心价值观，加强公民道德和精神文明建设。巩固提升全国文明城市创建成果，大力推进文明城市群建设。加强“中国诗歌之城”、“中国钢琴之城”、“读书之城”城市文化品牌建设。组织开展纪念抗战胜利70周年系列活动。深入实施国家公共文化服务体系示范区创建三年行动计划，完善文化基础设施，丰富文化产品供给，推进文化文艺精品生产，扎实开展文化惠民活动。有序推进广播电视“户户通、村村响”工程。办好2015全国青少年阳光体育大会。完成市体育场、市全民健身中心改造。加快推进三峡新闻中心建设和市滨江剧院改造。发挥文化体育公共设施作用，更好满足市民需求。加强网络文明传播，培育健康向上的网络文化。促进哲学社会科学、广播影视、新闻出版事业发展，繁荣文学艺术创作，加强文化遗产保护。加大教育投入，大力促进教育公平，加快推进教育现代化，努力建设“宜学之城”。实施特殊教育三年提升计划和第二期学前教育三年行动计划。巩固义务教育均衡发展成果，加快义务教育现代化学校创建，着力改善农村义务教育薄弱学校基本办学条件。推进高中教育多样化、特色化发展。围绕促进就业、产业转型升级，加快发展现代职业教育。加快推进市委党校、老年大学、青少年实践教育基地、妇女儿童活动中心、市一中新校区、夷陵中学新校区等重大项目建设。加快高等教育发展，支持三峡大学省部共建，融入地方建设；支持三峡大学科技学院和在宜高职院校加快发展；抓好三峡航空学院申报建设工作。加快推进市中心医院江南院区、市一医院、市三医院、三峡大学仁和医院、市妇女儿童医院、肿瘤医院等项目建设，完善城区医疗服务体系。加强乡镇卫生院、村卫生室和社区卫生服务中心建设，提升基层卫生服务能力。实施智慧医疗信息惠民工程，加快人口健康信息化建设。完善基层首诊、分级诊疗、双向转诊服务体系。加快推进全国健康城市创建工作。加强计划生育工作，促进人口长期均衡增长。全面开展地名普查。推进民族团结进步和援藏援疆工作。依法管理宗教事务。进一步抓好统计、档案、方志、科普、慈善、外事、侨务、港澳、对台、保密、消防、气象等各项工作。加强国防动员和后备力量建设，加大“双拥模范城”创建力度，巩固军政军民团结，推动军民融合发展。

　　提升社会治理水平。建立健全统筹城乡的网格化管理体系、广泛多层的社会化服务体系、协商共治的规范化自治体系和联通共享的信息化支撑体系，构建党委领导、政府负责、社会协同、公众参与、法治保障的社区治理格局，着力打造社会建设国家典范。推动葛洲坝集团社会职能逐步移交地方管理。创新有效预防和化解社会矛盾体制，建立畅通有序的诉求表达、矛盾调处机制。深化平安宜昌建设，加强群防群治工作，完善社会治安综合防控体系，依法防范和惩治违法犯罪活动。积极服务三峡工程安全运行。充分发挥工会、共青团、妇联、工商联、残联等群团组织在服务经济发展、创新社会治理中的重要作用。加强应急管理和防灾减灾体系建设。健全安全生产责任体系，强化安全生产监管执法，深入开展重点行业领域专项治理，创新安全监管方式，坚决杜绝重特大事故发生。

　　三、加快建设法治政府

　　深入贯彻依法治国基本方略，加快推进法治政府建设试点工作，把政府工作全面纳入法治轨道，用法治思维和法治理念推进改革、加快发展，努力建设职责科学、权责法定、执法严明、公开公正、高效廉洁、守法诚信的法治政府。在更高起点、更高水平上全面深化法治宜昌建设，加快建设全国同等城市依法治市示范区。

　　（一）深入推进依法行政。依法履行政府职能，切实做到“法定职责必须为、法无授权不可为”。落实常务会议集体学法制度，加强干部法制教育，提升干部法治能力。积极谋划地方立法工作。认真执行市人大及其常委会的各项决定和决议，主动听取人民政协的意见和建议，着力提高办理人大代表议案、建议和政协委员建议案、提案的质量和效率。支持法院、检察院依法独立公正行使审判权、检察权。严格执行重大行政决策程序规定，完善重大决策合法性审查机制，建立重大决策社会稳定风险第三方评估机制，确保行政决策制度科学、程序正当、过程公开、责任明确，不断提高依法、科学、民主决策水平。加大政务公开力度，完善政府信息发布和各类办事公开制度，稳步推进决策公开、执行公开、管理公开、服务公开、结果公开，重点推进财政预算、公共资源配置、重大建设项目批准和实施、社会公益事业建设等重点领域政府信息公开。加强制度廉洁性评估和规范性文件备案审查，高质量推进政府法制建设。深化行政复议和行政执法体制改革，建立完善综合执法体系。完善具体程序，规范操作流程，加强执法监督，不断提高行政执法水平。全面完成“六五”普法，健全普法宣传教育机制，增强全民法治观念，推进法治社会建设。

　　（二）着力提升政府效能。大兴“崇尚实干、狠抓落实”之风，全面推行一线工作法，构建抓落实责任链条，完善闭环督办落实制度体系，推动全市重大战略、重大决策、重点工作、重大项目的落实，确保执行政策不走样、不缩水，落实任务不打折扣、不出偏差。围绕改进服务态度、简化办事流程、提高服务效能，加快推进市、县、乡、村四级政务服务信息系统一体化。按照“四个一流”标准和要求，实施政务服务质量提升工程，积极开展政务服务综合标准化试点，创建一批政务服务品牌。建立政务公共服务质量满意度测评工作机制。严格执行行政机关首受责任制规定，进一步规范群众咨询、投诉受理工作。加强行政监察，加大治庸问责力度，坚决纠正不作为、乱作为，坚决克服懒政、怠政，坚决惩处失职、渎职。

　　（三）加强政府廉政建设。全面落实党风廉政建设政府党组主体责任，以正风肃纪为抓手，以制度约束为重点，持续推进政府系统党风廉政建设和反腐败工作，营造风清气正的经济社会发展环境。坚持纠正“四风”和树立新风并举，严格执行中央、省委、市委关于改进工作作风、密切联系群众各项规定，巩固和扩大群众路线教育实践活动成果。建立科学有效的权力运行制约和监督体系。严格执行“廉政清单”制度。继续深化廉政风险防控，加强审计监督，加快推进公共资金、国有资源、国有资产、经济责任审计全覆盖。在财政资金分配使用、国有资产监管、政府投资、政府采购、公共资源转让、公共工程建设、土地供应和矿产资源配置等重点领域和关键环节，排查廉政风险点，完善监管制度，建立常态化监督机制。深入推进预防和惩治腐败体系建设，支持纪检监察机关履行监督责任，严肃查处各类违法违纪案件，着力解决损害群众利益的突出问题。

　　各位代表！大潮澎湃处，激越看帆起。让我们在省委省政府和市委的坚强领导下，与全市人民一道，以更加饱满的热情、更加昂扬的斗志、更加务实的作风，开拓创新，竞进作为，向着建设“既大又强、特优特美”的现代化特大城市目标奋勇前进，为全省“建成支点、走在前列”作出新的更大贡献！
